# Sex Hygiene for the Young

**Published:** 1920-08-07

**Authors:** 


### Sex Hygiene for the Young.

### THE ROAD TO RACE-HEALTH.

The teaching of sex hygiene, said Miss M. Ewing Matheson at Birmingham, is still in the experimental stage. That is true, and it is a reproach to our civilisation that it should be so. It matters not what steps are taken by the Health Ministry or any voluntary or local organisation to combat venereal disease; no efforts will avail unless the importance of teaching sex hygiene at an early age is generally realised and practised upon a recognised plan. Fortunately, public opinion, and particularly parental opinion, is steadily losing its traditional prejudices in this matter, and beginning to understand the vital

**THE PUBLIC WELL-BEING**—(*continued*).

necessity, in the interest of the moral and physical well-being of the children of the race, of ensuring that they are not sent out into the world possessed of that ignorance which is romantically but falsely termed innocence, or with a dim and dangerous half-knowledge which excites the worst kind of prurient curiosity.

At present the chief difficulty seems to be a practical one, though we think it is greatly magnified. In what form, it is asked, can such teaching be given so as to create the right impression and avoid confusion and mistakes? Undoubtedly the right place for systematic training is the school; and though it may be quite true that a proportion of school teachers of both sexes are unfitted temperamentally or for other reasons to impart knowledge in a form that shall be above criticism, it should be a comparatively easy matter to regularise the teaching of sex hygiene as a part of the school curriculum on a basis as simple and progressive as the teaching of literature or history or natural science. If the data and the method are supplied officially upon scientific but intelligible lines, a teacher of average intelligence will be able to make the same efficient use of the instructions that he would do in the case of any other school subject.

It must not be forgotten, of course, that before the school age the teacher's task can be immensely simplified by home education. Here it is that the tact and common sense of the parent can and should play a great part. The golden rule, implied in a most interesting address delivered by Mrs. C. Guest, from the working mother's point of view, is to answer a child's questions honestly and straightforwardly and in plain language, without any attempt at suggesting a mystery or concealing a fact. In ninety-nine cases out of a hundred a child's spontaneous questions will open the way. It is good for a child, for instance, to learn early the reason for a mother's great love; and the reply to those persons who fear the consequences of chatter among children on the subject of sex is the almost invariable experience that when children are told the truth about anything they do not bother about it any more.

It is for the parents, too, to exercise their responsibility when children approach the dangerous adolescent age. Countless disasters would have been avoided in the past if at this difficult and emotional time parents had the courage and the wisdom to explain the changes taking place and the responsibilities in connection with them, and to give the warning of possible temptations. "The adolescent can be told of the need to keep a clean, healthy body in readiness for the time when marriage is desired—the harm that is done to the children of the future if an unhealthy life is led." We look forward to the time when it will be no more necessary to insist on the need for sex hygiene than to expatiate on the advantages of compulsory education.

